# Oxidative Stress and Metabolic Reprogramming in Osteoclastogenesis: Molecular Mechanisms and Antioxidant Therapy for Bone Disorders

**DOI:** 10.3390/antiox15101305

**Published:** 2026-10-09

**Authors:** Mohammad Ibtehaz Alam, Fatima Farhana, Tajiba Rahman Khan, Farjana Sharmin, Mst Hurunnaher, Yu Katsuma, Eiko Sakai, Yoshinori Sumita

**Affiliations:** 1 Department of Periodontology & Oral Pathology, University Dental College, Dhaka 1217, Bangladesh; 2 Department of Prosthodontics, University Dental College, Dhaka 1217, Bangladesh; fatimafarhana.2011@gmail.com; 3 Department of Gynecology and Obstetrics, Green Medical College and Hospital, Dhaka 1205, Bangladesh; rahman.tajiba@gmail.com; 4 Department of Dental Public Health, University Dental College, Dhaka 1217, Bangladesh; farjana222@gmail.com (F.S.); hira.bd03@gmail.com (M.H.); 5 Department of Medical Research and Development for Oral Disease, Nagasaki University Graduate School of Biomedical Sciences, Nagasaki 852-8588, Japan; bb55324203@ms.nagasaki-u.ac.jp (Y.K.); y-sumita@nagasaki-u.ac.jp (Y.S.)

**Keywords:** oxidative stress, osteoclast metabolism, reactive oxygen species, antioxidants, Nrf2, mitochondrial dysfunction, NFATc1, bone remodeling, osteoporosis, arthritis

## Abstract

Osteoclasts are the primary bone-resorbing cells responsible for maintaining skeletal homeostasis through balanced bone remodeling. Accumulating evidence suggests that oxidative stress is an emerging regulator of osteoclast differentiation, metabolic reprogramming, and bone resorption, arising from excessive reactive oxygen species (ROS) and insufficient antioxidant defenses. Dysregulated redox signaling contributes to osteoporosis, rheumatoid arthritis, periodontitis, periapical lesions, and other osteolytic disorders. This review summarizes recent advances in the molecular mechanisms linking oxidative stress to osteoclast metabolism, focusing on ROS sources, mitochondrial dysfunction, redox-sensitive signaling pathways, endogenous antioxidant systems, and emerging antioxidant-based therapies. Oxidative stress regulates osteoclastogenesis through interconnected signaling networks, including NF-κB, MAPKs, PI3K/Akt, AMPK, mTOR, NFATc1, and Keap1/Nrf2. Under both physiological and pathological conditions, osteoclast function is regulated by mitochondrial dynamics, mitophagy, and epigenetic mechanisms. Although both synthetic and natural antioxidants have been shown to reduce pathological osteoclast activity in preclinical studies, limitations in bioavailability, target specificity, and long-term efficacy hinder clinical translation. Advances in multi-omics technologies, single-cell analyses, biomarker discovery, precision medicine, and targeted antioxidant delivery may facilitate the development of effective therapies for oxidative stress-related bone disorders.

## 1. Introduction

Bone remodeling is a continuous process that maintains mineral homeostasis, repairs microdamage, and protects skeletal integrity through the coordinated action of osteoclasts, osteoblasts, and osteocytes [1,2,3]. Disruption of this coupling leads to excessive bone loss and skeletal deterioration in conditions such as osteoporosis, rheumatoid arthritis (RA), periodontitis, peri-implantitis, periapical lesions, and osteolytic bone metastases [4,5,6]. Among the mechanisms underlying these conditions, oxidative stress has been identified as a key factor that accelerates osteoclast differentiation and contributes to pathological bone loss.

Osteoclasts are multinucleated cells that develop from monocyte/macrophage precursors upon stimulation with macrophage colony-stimulating factor (M-CSF) and receptor activator of nuclear factor-κB (RANK) ligand (RANKL). RANKL binding to RANK triggers multiple intracellular cascades that culminate in the activation of nuclear factor of activated T cell 1 (NFATc1), the master transcription factor controlling osteoclast differentiation and function [7,8,9,10]. Mature osteoclasts enable the acidification and degradation of mineralized bone matrix through specialized structures, such as the sealing zone and ruffled border [7,11]. To meet the high bioenergetic demands of bone resorption, osteoclast differentiation necessitates extensive metabolic reprogramming that extends beyond classical cytokine signaling and is characterized by increased glycolysis, mitochondrial oxidative phosphorylation, glutamine utilization, and lipid metabolism [12,13,14,15].

Reactive oxygen species (ROS) are essential signaling molecules that regulate immune responses, cell differentiation, and proliferation under physiological conditions. However, excessive ROS production or insufficient antioxidant defenses disrupt cellular redox homeostasis, resulting in oxidative stress that damages macromolecules, impairs mitochondrial function, and promotes inflammatory signaling [16,17,18,19]. Nicotinamide adenine dinucleotide phosphate (NADPH) oxidases, mitochondrial respiration, and other oxidase systems are major sources of ROS, including superoxide anions, hydrogen peroxide, and hydroxyl radicals [16,17,20]. A growing body of evidence suggests that oxidative stress influences iron metabolism, autophagy, mitochondrial function, inflammatory responses, and several redox-sensitive pathways, thereby contributing to osteoclastogenesis [15,17,21,22,23,24,25]. Therefore, maintaining redox homeostasis is crucial for ongoing physiological bone remodeling.

The Kelch-like ECH-associated protein 1 (Keap1)/nuclear factor erythroid 2-related factor 2 (Nrf2) signaling pathway has been identified as a key endogenous defense system against oxidative stress. Under basal conditions, Keap1 sequesters Nrf2 and directs it toward proteasomal degradation. Oxidative stress disrupts this interaction, enabling Nrf2 to translocate to the nucleus and induce the expression of cytoprotective and antioxidant genes that restore redox balance [26,27,28,29,30]. In experimental models of osteoporosis, Nrf2 activation attenuated oxidative stress, inhibited osteoclast differentiation, and reduced pathological bone resorption in mice. However, recent studies suggest that rather than completely eliminating ROS, therapeutic approaches should aim to restore redox homeostasis, given that physiological ROS are also necessary for normal osteoclastogenesis. Furthermore, recent developments have expanded our understanding of osteoclast biology by revealing the important roles of mitochondrial dynamics, metabolic adaptation, epigenetic regulation, and non-coding RNAs (ncRNAs) in oxidative stress-mediated bone remodeling. Meanwhile, CRISPR-based functional genomics, single-cell transcriptomics, spatial transcriptomics, and multi-omics techniques have uncovered previously unrecognized osteoclast heterogeneity and identified new molecular targets implicated in pathological bone resorption [15,31,32,33,34]. These innovations are reshaping our understanding of redox regulation in skeletal biology and creating new possibilities for precision medicine.

Despite these developments, several challenges remain to be addressed. Most mechanistic evidence has been derived from experimental models, and pharmacokinetic limitations, poor target specificity, poor bioavailability of antioxidant compounds, and a lack of robust clinical trials have impeded clinical translation [17,30,35]. Inconsistencies between studies may reflect differences in disease models and methods used to assess oxidative stress. Integrating redox biology, osteoimmunology, bone metabolism, advanced omics technologies, and precision medicine is essential for overcoming these constraints. In this review, we integrate recent advances in understanding the role of oxidative stress in osteoclast metabolism and bone resorption, with an emphasis on ROS production, metabolic reprogramming, redox-sensitive signaling pathways, endogenous antioxidant systems, epigenetic regulation, and antioxidant-based therapeutic approaches. Additionally, we outline current limitations, emerging technologies, and potential strategies for developing precise antioxidant therapies for bone disorders associated with oxidative stress.

## 2. Osteoclast Biology

Osteoclasts, the sole cells responsible for physiological bone resorption, are highly specialized multinucleated cells derived from hematopoietic monocyte/macrophage precursors. Through ongoing bone remodeling, osteoclasts interact with osteoblasts and osteocytes to preserve skeletal homeostasis. M-CSF and RANKL are the primary regulators of osteoclast differentiation. RANKL initiates signaling cascades that converge on NFATc1, whereas M-CSF promotes precursor survival, proliferation, and RANK receptor expression [7,8,9,10]. NFATc1 activation induces osteoclast-specific genes, including tartrate-resistant acid phosphatase (TRAP), cathepsin K (CTSK), dendritic cell-specific transmembrane protein (DC-STAMP), ATP6V0D2, and calcitonin receptor (CTR). Taken together, these genes control cell fusion, cytoskeletal organization, and bone resorption.

The maturation of osteoclast precursors, which fuse to form multinucleated cells with specialized structures such as the sealing zone and ruffled border, enables acidification of the resorption lacuna and enzymatic breakdown of the mineralized bone matrix [7,11]. Although osteoclastogenesis depends on cytokine-mediated signaling, growing evidence suggests that differentiation and activation also require substantial metabolic reprogramming. To meet the high energy and biosynthetic demands associated with multinucleation, cytoskeletal remodeling, proton secretion, and matrix degradation, osteoclasts undergo coordinated changes in glucose, lipid, amino acid, and mitochondrial metabolism. In addition to producing the required adenosine triphosphate (ATP), these metabolic pathways produce intermediates that modulate redox homeostasis, intracellular signaling, and epigenetic modifications. This establishes cellular metabolism as a key regulator of osteoclast differentiation and function, extending beyond its role in energy production [12,13,14,15].

## 3. Metabolic Reprogramming During Osteoclastogenesis

Metabolic reprogramming has emerged as a fundamental regulator of osteoclast differentiation and function rather than merely a consequence of cellular activation (Table 1). Recent transcriptomic and metabolomic analyses have revealed dynamic reprogramming of glucose, lipid, amino acid, and mitochondrial metabolism during osteoclast differentiation, underscoring metabolism as an active determinant of osteoclast fate and bone-resorptive capacity that extends beyond energy production [13,14,15,36].

### 3.1. Glycolysis and Glucose Metabolism

During osteoclast differentiation, glycolysis rapidly increases to provide ATP and the metabolic intermediates required for precursor fusion and bone resorption. According to recent in vitro studies, RANKL stimulation increases the expression of important glycolytic enzymes, such as hexokinase 2 (HK2), phosphofructokinase (PFK), and pyruvate kinase M2 (PKM2), thereby accelerating glycolytic flux and increasing glucose uptake through the induction of glucose transporters, especially glucose transporter type 1 (GLUT1) [36,37,38,39]. In addition to ATP production, glycolytic intermediates support the biosynthesis of lipids, amino acids, and nucleotides required for rapid cell division. Glycolytic metabolism was found to be essential for osteoclastogenesis rather than merely a consequence of differentiation, as pharmacological inhibition of glycolysis markedly suppressed osteoclast differentiation, reduced the formation of TRAP-positive multinucleated cells, and attenuated bone-resorptive activity. Similarly, recent animal studies have demonstrated that inhibition of glycolytic enzymes reduces pathological bone loss, whereas increased glycolytic activity is associated with enhanced osteoclast formation in inflammatory bone diseases [37,38]. These findings indicate that glycolysis is a crucial metabolic pathway linking RANKL signaling to osteoclast differentiation.

### 3.2. Mitochondrial Oxidative Phosphorylation and Bioenergetics

During osteoclast maturation, mitochondrial oxidative phosphorylation (OXPHOS) is the main source of sustained ATP production required for proton transport, cytoskeletal organization, and extracellular matrix degradation. Recent evidence suggests that the c-Myc–PGC-1β signaling axis plays a central role in driving the increased mitochondrial biogenesis, respiratory capacity, and oxidative phosphorylation that accompany osteoclast differentiation [13,15,40]. Mitochondrial respiration is a pivotal source of intracellular ROS and is crucial for osteoclast function. Excessive ROS levels can cause mitochondrial dysfunction and oxidative damage, thereby compromising osteoclast homeostasis, whereas moderate ROS levels act as secondary messengers that enhance RANKL-dependent signaling and promote physiological NFATc1 activation [21,25,40]. Consequently, mitochondrial quality control mechanisms, including mitophagy, fusion–fission dynamics, and mitochondrial biogenesis, are increasingly recognized as crucial for maintaining physiological osteoclast activity and mitochondrial integrity.

### 3.3. Fatty Acid and Amino Acid Metabolism

Recent metabolomic studies suggest that osteoclast differentiation is supported by metabolic substrates other than glucose. Fatty acid oxidation (FAO) plays a key role in ATP production, particularly under nutrient-limited conditions or during prolonged osteoclast activation [13,41]. FAO supports mitochondrial oxidative metabolism and glycolysis by supplying acetyl-CoA to the tricarboxylic acid (TCA) cycle. Similarly, osteoclast bioenergetics and biosynthesis depend heavily on glutamine metabolism. In vitro studies have shown that glutamine maintains intracellular redox balance during differentiation by replenishing TCA cycle intermediates through anaplerosis, supporting nucleotide synthesis, and amino acid and glutathione biosynthesis [14,42]. These findings suggest that the coordinated use of glucose, fatty acids, and amino acids provides the metabolic flexibility required to maintain osteoclast function under various physiological and pathological conditions.

### 3.4. Metabolic Plasticity and Microenvironmental Adaptation

Osteoclast metabolism is highly dynamic and varies depending on the stage of development and the microenvironment. According to recent studies, osteoclasts shift between OXPHOS, glycolysis, and other fuel sources in response to oxidative stress, inflammatory cytokines, oxygen tension, and nutrient availability [15,41]. Nutrient-sensing pathways that integrate extracellular stimuli with intracellular metabolic programs, such as adenosine monophosphate (AMP)-activated protein kinase (AMPK), mechanistic target of rapamycin (mTOR), hypoxia-inducible factor-1α (HIF-1α), and sirtuin signaling, contribute to the regulation of metabolic plasticity. In animal models of osteoporosis, inflammatory arthritis, diabetes, and aging, osteoclasts undergo substantial metabolic remodeling, characterized by altered mitochondrial function, elevated glycolysis, and disrupted redox homeostasis [18,25,41]. Under stressful circumstances, these adaptive responses allow osteoclasts to maintain their bone-resorptive activity. However, when metabolic regulation is dysregulated, these adaptations may also contribute to pathological bone loss.

### 3.5. Metabolic Reprogramming as a Therapeutic Target

Current evidence suggests that metabolic reprogramming is a key regulator of osteoclast survival, activation, and differentiation. During osteoclastogenesis, glycolysis, OXPHOS, FAO, and amino acid metabolism function as interrelated metabolic networks that regulate energy production, biosynthesis, and redox homeostasis. Nevertheless, the relative contributions of individual metabolic pathways appear to vary according to developmental stage, disease context, and microenvironment, highlighting the complexity of osteoclast metabolic regulation. Recent developments in metabolomics, CRISPR-based functional genomics, spatial transcriptomics, stable isotope tracing, and single-cell RNA sequencing have begun to define stage-specific metabolic programs and reveal previously unknown therapeutic targets [31,32,33,34]. Combining these advanced technologies with redox biology is expected to yield a more comprehensive understanding of osteoclast metabolism and accelerate the development of metabolism-based therapeutic approaches for oxidative stress-associated osteolytic disorders.

## 4. Sources of Oxidative Stress in Osteoclasts

ROS are continuously generated during cellular metabolism and function as essential secondary messengers that regulate cell proliferation, differentiation, apoptosis, and immune responses. Physiological ROS levels in osteoclasts are essential for normal RANKL-induced osteoclast differentiation and bone remodeling. However, excessive ROS production or insufficient antioxidant defenses can disrupt redox homeostasis, resulting in oxidative stress that accelerates bone resorption, promotes osteoclastogenesis, and causes pathological bone loss. Numerous intracellular and microenvironmental factors, including NADPH oxidases (NOXs), mitochondrial dysfunction, endoplasmic reticulum (ER) stress, iron dysregulation, inflammatory cytokines, hypoxia, aging, metabolic disorders, cigarette smoking, and chronic inflammatory diseases such as periodontitis, have been implicated in oxidative stress in osteoclasts [43,44,45,46]. Under pathological conditions, these pathways interact to form an integrated redox network that enhances osteoclast activation rather than functioning independently (Table 2).

### 4.1. NADPH Oxidases: The Primary Enzymatic Source of ROS

The NOX family is the primary enzymatic source of ROS among intracellular ROS-generating systems during osteoclastogenesis. Recent studies have demonstrated that NOX1, NOX2, and NOX4 are expressed during osteoclast differentiation. NOX4 supports osteoclast maturation and bone resorption, whereas NOX1 and NOX2 regulate early precursor commitment [43,44]. Nuclear factor kappa B (NF-κB), mitogen-activated protein kinase (MAPK), and NFATc1 signaling pathways are amplified by NOX-dependent ROS production following RANKL stimulation. Pharmacological inhibition or genetic deletion of NOX isoforms markedly reduces intracellular ROS accumulation, suppresses osteoclast formation, and protects against inflammatory and osteoporotic bone loss, as observed in animal studies [44,45,46]. These findings highlight NOX enzymes as promising therapeutic targets for oxidative stress-associated skeletal diseases.

### 4.2. Mitochondrial ROS and Bioenergetic Stress

Mitochondria are another important source of intracellular ROS during osteoclast differentiation. To meet the high energy demands of proton secretion, cytoskeletal remodeling, and matrix degradation, osteoclast maturation is accompanied by increased mitochondrial biogenesis and OXPHOS, as discussed in the previous section. Recent mechanistic evidence suggests that increased mitochondrial respiration can increase mitochondrial ROS production, which promotes RANKL-induced signaling under physiological circumstances but becomes harmful when ROS accumulate excessively [21,40,47,48]. Overproduction of mitochondrial ROS accelerates pathological bone resorption by impairing respiratory function, disrupting the mitochondrial membrane potential, promoting mitochondrial DNA damage, and amplifying inflammatory signaling. Therefore, mitochondrial quality control mechanisms, such as mitophagy, fusion–fission dynamics, and mitochondrial biogenesis, are considered crucial for preserving bioenergetic homeostasis and limiting oxidative damage [40,47].

### 4.3. ER Stress and Iron Dysregulation

Recent research suggests that the ER also contributes to oxidative stress during osteoclastogenesis. Prolonged ER stress triggers the unfolded protein response (UPR), which disrupts calcium homeostasis, increases ROS production, and activates inflammatory signaling pathways that promote osteoclast differentiation [46,49,50]. Although brief ER stress promotes adaptive cellular responses, prolonged activation exacerbates oxidative damage and disrupts cellular homeostasis [46]. Iron metabolism is another key factor regulating osteoclast redox status. Several animal and human studies have shown that iron overload increases osteoclast differentiation, accelerates bone loss in osteoporosis and inflammatory bone diseases, and promotes ROS generation via Fenton chemistry [51,52]. Ferroptosis, a type of cell death driven by iron-dependent lipid peroxidation, has recently emerged as a possible modulator of skeletal remodeling [49,52]. Despite mounting evidence that ferroptosis-related pathways affect osteoclast function through interactions among iron metabolism, lipid peroxidation, and oxidative stress, their precise role in osteoclast biology remains unclear.

### 4.4. Inflammatory and Microenvironmental Drivers of Oxidative Stress

In addition to intracellular ROS-producing systems, pathological microenvironmental factors substantially increase oxidative stress during osteoclastogenesis. Pro-inflammatory cytokines, such as tumor necrosis factor (TNF)-α, interleukin (IL)-1β, IL-6, and IL-17, interact with RANKL to enhance ROS production, trigger osteoclastogenic signaling pathways, and impair endogenous antioxidant defenses. This interaction contributes to a vicious cycle of inflammation and bone loss [18,21,53]. Hypoxia increases mitochondrial ROS levels during prolonged oxygen deprivation and further stimulates metabolic remodeling via HIF-1α. Likewise, cellular senescence, reduced antioxidant capacity, and mitochondrial dysfunction are hallmarks of aging that contribute to a pro-oxidant microenvironment that promotes osteoclast activation [25,41,54].

Environmental exposures and metabolic disorders exacerbate oxidative stress. Chronic hyperglycemia increases ROS production through mitochondrial dysfunction, advanced glycation end-product formation, and persistent inflammation, thereby enhancing osteoclast activity in diabetes mellitus [55,56]. Periodontal pathogens promote ROS production and pro-inflammatory cytokine release, thereby increasing RANKL expression and accelerating alveolar bone destruction, while cigarette smoking introduces exogenous oxidants that exacerbate oxidative stress and inflammatory signaling [57,58].

Collectively, these findings indicate that oxidative stress in osteoclasts results from the coordinated interaction of pathological microenvironmental stimuli and intracellular ROS-generating systems, rather than from a single source. This integrated understanding may provide new opportunities for developing precise antioxidant therapies that selectively suppress pathological bone resorption while preserving physiological skeletal remodeling.

## 5. Redox Signaling Networks Regulating Osteoclastogenesis

**Figure 1:**
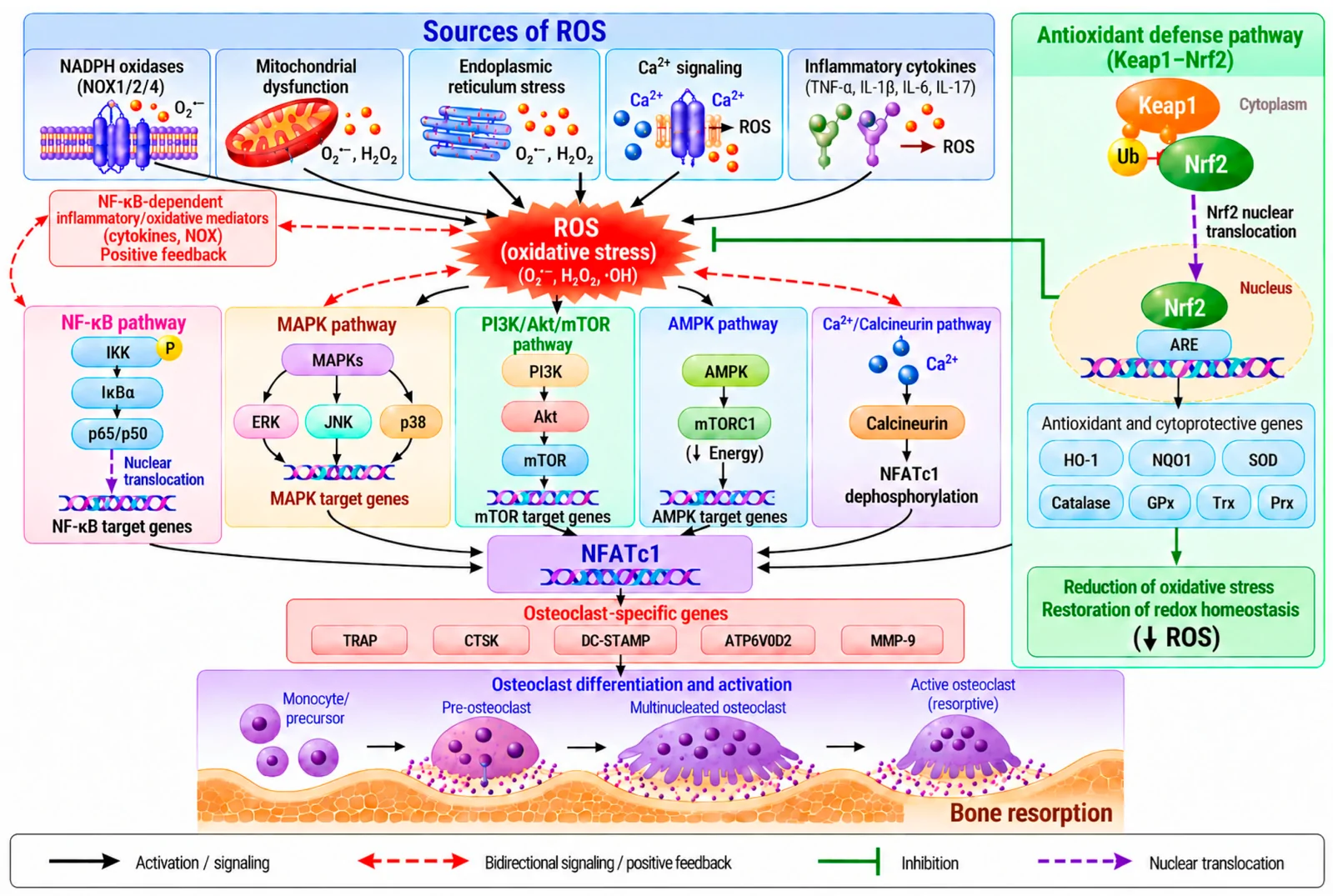
**ROS-mediated signaling and antioxidant defense in osteoclasts.** Reactive oxygen species (ROS) are generated through NADPH oxidases (NOX1/2/4), mitochondrial dysfunction, endoplasmic reticulum stress, Ca^2+^ signaling, and inflammatory cytokines. ROS interact with NF-κB-dependent inflammatory/oxidative mediators through a positive-feedback mechanism and engage the MAPK, PI3K/Akt/mTOR, AMPK, and Ca^2+^/calcineurin pathways. These signaling pathways converge on nuclear factor of activated T cells 1 (NFATc1), promoting the expression of osteoclast-specific genes, including TRAP, CTSK, DC-STAMP, ATP6V0D2, and MMP-9, thereby supporting osteoclast differentiation and activation and ultimately bone resorption. In parallel, the Keap1–Nrf2 antioxidant defense pathway promotes Nrf2 nuclear translocation and activation of antioxidant and cytoprotective genes, including HO-1, NQO1, SOD, catalase, GPx, Trx, and Prx, contributing to the reduction in oxidative stress and restoration of redox homeostasis. Black arrows indicate activation/signaling, red dashed double-headed arrows indicate bidirectional signaling/positive feedback, green T-bars indicate inhibition, and purple dashed arrows indicate nuclear translocation.

ROS are considered vital intracellular signaling molecules rather than merely detrimental metabolic byproducts. During RANKL-induced osteoclastogenesis, physiological ROS function as secondary messengers that modulate signaling pathways involved in differentiation, metabolism, and bone resorption. However, excessive ROS disrupt redox homeostasis, resulting in sustained activation of osteoclastogenic signaling and pathological bone loss. ROS orchestrate an interconnected network of redox-sensitive pathways, including NF-κB, MAPKs, PI3K/Akt, mTOR, AMPK, calcium/calcineurin, NFATc1, cAMP response element-binding protein (CREB), c-Fos, forkhead box O (FOXO), and Keap1/Nrf2 (Figure 1), which collectively integrate inflammatory, metabolic, and oxidative signals to regulate osteoclast formation and function, as demonstrated in recent in vitro and animal studies [2,18,35,43].

### 5.1. NF-κB Signaling

The NF-κB pathway is one of the earliest and most critical signaling cascades activated during osteoclast differentiation [57]. Recent mechanistic studies have demonstrated that physiological ROS generated by NADPH oxidases and mitochondria function as secondary messengers that facilitate NF-κB activation, whereas sustained oxidative stress prolongs NF-κB activation and accelerates pathological bone resorption [18,43]. Moreover, the relationship between the ROS signaling pathway and the NF-κB signaling pathway is not one-sided. ROS can be further stimulated by mediators that have been activated due to NF-κB signaling, such as cytokines and NADPH oxidases, thus creating a feedback loop. RANKL binding to RANK activates tumor necrosis factor receptor-associated factor 6 (TRAF6), which then activates transforming growth factor-β-activated kinase 1 (TAK1) and the IκB kinase (IKK) complex, leading to IκB degradation and nuclear translocation of the p65/p50 NF-κB heterodimer [59]. Following the induction of c-Fos and NFATc1 expression, activated NF-κB promotes expression of osteoclast-specific genes, such as TRAP, CTSK, DC-STAMP, MMP-9, and ATP6V0D2 [60,61]. Further in vivo studies have demonstrated that osteoclast formation and inflammatory bone loss are markedly reduced by pharmacological inhibition of NF-κB or suppression of intracellular ROS. It is important to emphasize that therapeutic strategies should restore redox balance rather than eliminate ROS signaling, as complete NF-κB blockade or indiscriminate ROS removal may impair physiological bone remodeling and immune function [2].

### 5.2. MAPK Signaling Pathways

Oxidative stress is transduced into transcriptional programs that regulate osteoclast differentiation via MAPK signaling. Precursor proliferation, survival, cytoskeletal remodeling, and osteoclast maturation are all coordinated by ROS through redox-sensitive kinase cascades, as demonstrated in experimental studies [62]. While p38 stimulates NFATc1 expression and osteoclast-specific gene transcription, JNK activates c-Jun and AP-1, whereas ERK mainly supports cell survival. [62,63]. Interestingly, osteoclastogenesis is consistently suppressed by MAPK inhibition; however, the relative contribution of each MAPK pathway varies depending on the microenvironment and disease context [63,64].

### 5.3. PI3K/Akt/mTOR and AMPK: Linking Redox Signaling with Metabolism

ROS also modulate the nutrient-sensing pathways that link osteoclast differentiation to metabolism. Recent studies have shown that activation of the PI3K/Akt/mTOR axis promotes osteoclast formation by enhancing glycolysis, mitochondrial activity, protein synthesis, and cell survival [35,36]. During oxidative and energy stress, AMPK serves as a metabolic checkpoint that limits excessive anabolic activity. Osteoclasts can integrate intracellular energy with redox signaling through crosstalk between these pathways, thereby facilitating metabolic adaptation during bone resorption and differentiation [65,66].

### 5.4. Calcium Signaling and NFATc1 Activation

Calcium signaling is a major downstream convergence point of redox-sensitive signaling pathways. Previous studies have shown that calcineurin, which dephosphorylates NFATc1 and promotes its nuclear translocation, is activated by ROS-induced calcium oscillations [43]. NFATc1 promotes multinucleation and bone-resorptive activity by inducing the expression of osteoclast-specific genes, such as TRAP, CTSK, DC-STAMP, ATP6V0D2, and MMP-9, in conjunction with c-Fos, AP-1, and CREB [59,60].

IRF8 is a well-established negative regulator of osteoclast differentiation. Suppression of Irf8 expression promotes the induction of NFATc1, the master transcription factor of osteoclastogenesis [67]. Subsequent studies demonstrated that RANKL-RANK signaling induces ROS production, which enhances mitochondrial biogenesis through PGC-1β [68]. Furthermore, increased mitochondrial biogenesis suppresses Irf8 expression via DNA methylation [69]. Taken together, these findings indicate that RANKL-RANK signaling promotes ROS production, leading to enhanced mitochondrial biogenesis and epigenetic suppression of Irf8, thereby relieving the inhibitory effect of IRF8 on NFATc1 expression and facilitating osteoclast differentiation.

### 5.5. Integrated Redox Signaling Networks and Therapeutic Perspectives

To regulate osteoclast differentiation and bone resorption, ROS coordinate the activities of NF-κB, MAPKs, PI3K/Akt/mTOR, AMPK, calcium signaling, and antioxidant pathways that converge on the regulation of NFATc1. Instead of operating independently, redox-sensitive pathways form interconnected signaling networks that integrate oxidative, inflammatory, and metabolic signals.

In preclinical studies, natural antioxidants, such as curcumin, resveratrol, quercetin, epigallocatechin gallate (EGCG), and sulforaphane, have been shown to suppress osteoclastogenesis by reducing oxidative stress, inhibiting NF-κB and MAPK signaling, and activating Nrf2-mediated antioxidant defenses [70,71,72,73]. Recently, Yang et al. demonstrated that *Spirulina platensis*, a naturally occurring microorganism used as a food ingredient, exerted potent therapeutic effects against RA by activating Nrf2 and suppressing the protein levels of NFATc1, c-Fos, and cathepsin K [74]. Despite these encouraging results, limitations in bioavailability and target specificity, as well as a lack of long-term clinical evidence, hinder the clinical translation of identified natural antioxidants. Further research is required to better understand the spatiotemporal regulation of redox signaling and to develop targeted antioxidant therapies for osteolytic diseases.

## 6. Nrf2 and Endogenous Antioxidant Systems

### 6.1. FOXO–Nrf2 Crosstalk and Endogenous Antioxidant Signaling

Major endogenous antioxidant systems that limit excessive ROS accumulation include the Keap1/Nrf2 pathway and FOXO transcription factors. According to recent mechanistic and animal studies, FOXO and Nrf2 activation limits pathological osteoclast activation by upregulating antioxidant enzymes, maintaining mitochondrial integrity, and reducing oxidative damage [26,27,28,29,30,66,73]. However, therapeutic modulation of antioxidant signaling should maintain basal redox signaling while preventing chronic oxidative stress because physiological ROS are necessary for normal osteoclastogenesis.

### 6.2. Keap1–Nrf2 Signaling in Redox Homeostasis

Balanced bone remodeling requires preservation of intracellular redox equilibrium. Excessive ROS triggers inflammatory signaling, mitochondrial dysfunction, and pathological bone resorption, although physiological levels of ROS are necessary for RANKL-induced osteoclast differentiation [18,75]. Recent in vitro and animal studies suggest that the Keap1–Nrf2 pathway is a key regulator of endogenous antioxidant defense during osteoclastogenesis [76,77]. Under physiological conditions, Keap1 sequesters Nrf2 in the cytoplasm, thereby facilitating its ubiquitination and proteasomal degradation. Oxidative or electrophilic stress disrupts the Keap1–Nrf2 complex, allowing Nrf2 to translocate to the nucleus, bind to antioxidant response elements, and induce the expression of several cytoprotective and antioxidant genes [76,78].

### 6.3. Nrf2-Regulated Antioxidant Enzymes

Heme oxygenase-1 (HO-1), NAD(P) quinone oxidoreductase-1 (NQO1), superoxide dismutase (SOD), catalase, glutathione peroxidase (GPx), thioredoxin (Trx), and peroxiredoxins (Prxs) are among the antioxidant enzymes and redox-regulatory proteins induced by Nrf2 activation [79,80,81]. Collectively, these effectors contribute to the regulation of redox-sensitive signaling pathways involved in osteoclast differentiation, limit excessive ROS accumulation, and maintain mitochondrial homeostasis. Among these effectors, HO-1 has been identified as a crucial mediator of Nrf2-dependent skeletal protection. Recent mechanistic studies have shown that HO-1 reduces inflammatory cytokine production, suppresses NF-κB activation, and inhibits RANKL-induced osteoclastogenesis. Taken together, the NQO1, SOD, catalase, GPxs, and Trx/Prx systems help preserve physiological ROS signaling and protect against oxidative damage [72,78,80,81].

### 6.4. Role of Nrf2 in Osteoclastogenesis and Bone Diseases

In animal models of osteoporosis, RA, periodontitis, and diabetic bone disease, experimental evidence consistently shows that activation of the Keap1–Nrf2 pathway suppresses intracellular ROS accumulation, maintains mitochondrial function, inhibits NF-κB and MAPK signaling, and attenuates osteoclast differentiation [18,81,82]. Conversely, Nrf2 deficiency compromises skeletal homeostasis by increasing oxidative stress, promoting osteoclastogenesis, and accelerating bone loss, thereby highlighting the crucial role of Nrf2 in maintaining redox homeostasis [77,78,82]. Physiological ROS are essential for normal osteoclast differentiation. Therefore, therapeutic modulation targeting Nrf2 should aim to restore redox balance rather than completely suppress ROS signaling.

Nrf2 is a promising therapeutic target for oxidative stress-related bone diseases owing to its crucial role in endogenous antioxidant defense. Preclinical studies have demonstrated that natural antioxidants, including sulforaphane, curcumin, resveratrol, quercetin, EGCG, and melatonin, suppress osteoclastogenesis by activating Nrf2 signaling while inhibiting NF-κB-mediated inflammation [71,72,73,81]. New approaches, such as targeted antioxidant delivery systems and synthetic Nrf2 activators, may enhance therapeutic efficacy. However, limited long-term clinical evidence, pharmacokinetic variability, and poor bioavailability remain major barriers to their clinical translation.

## 7. Epigenetic and Non-Coding RNA Regulation of Oxidative Stress in Osteoclastogenesis

Epigenetic regulation has emerged as a critical mechanism that links oxidative stress to osteoclast differentiation and pathological bone resorption. Unlike genetic changes, epigenetic modifications control gene expression without altering the DNA sequence, allowing osteoclasts to adapt rapidly to metabolic, oxidative, and inflammatory stresses. According to recent transcriptomic, epigenomic, and functional studies, DNA methylation, histone modifications, RNA methylation, and ncRNAs interact extensively with redox-sensitive signaling pathways, such as NF-κB, MAPKs, PI3K/Akt, AMPK, and Nrf2, to regulate osteoclast metabolism, differentiation, and survival. Dysregulation of these epigenetic and non-coding RNA mechanisms has been implicated in the pathogenesis of RA, periodontitis, osteoporosis, periapical lesions, and other osteolytic disorders.

### 7.1. ncRNAs Regulating Osteoclast Redox Homeostasis

ncRNAs are among the most well-studied post-transcriptional and epigenetic regulators of osteoclastogenesis. Numerous microRNAs (miRNAs) that influence osteoclast differentiation by targeting elements of the RANKL-RANK signaling cascade, such as TRAF6, NF-κB, c-Fos, NFATc1, and Nrf2, have been identified in recent studies [83,84,85]. miRNAs can stimulate or inhibit osteoclast formation and bone resorption depending on their downstream targets. Competing endogenous RNAs (ceRNAs) can sequester miRNAs, thereby modulating oxidative stress, mitochondrial function, inflammatory signaling, and antioxidant responses. Long non-coding RNAs (lncRNAs) and circular RNAs also contribute to the complexity of redox regulation [84,85,86]. Recent studies suggest these ncRNA-mediated regulatory networks help osteoclasts adapt to physiological and pathological conditions by fine-tuning intracellular redox homeostasis.

### 7.2. DNA, Histone, and RNA Modifications

Chromatin remodeling provides an additional layer of redox regulation to ncRNA activity during osteoclastogenesis. Recent research has shown that oxidative stress alters DNA methylation patterns and the activity of histone-modifying enzymes, causing transcriptional changes that affect osteoclast-specific genes, inflammatory responses, and antioxidant defenses [87,88]. Histone acetylation and methylation regulate chromatin accessibility, thereby modulating the expression of NFATc1, c-Fos, and other transcription factors required for osteoclast differentiation. In addition, epigenetic regulation is an important component of skeletal biology. N6-methyladenosine (m6A), which controls RNA stability, splicing, translation, and degradation, is the most abundant internal mRNA modification. Recent mechanistic studies have indicated that m6A methylation integrates cellular metabolism, redox signaling, and osteoclast differentiation, representing an emerging area of research in bone biology, although the relationship between m6A and oxidative stress remains unclear [89].

### 7.3. Crosstalk Between Epigenetics and Redox Signaling

Epigenetic mechanisms interact closely with traditional redox-sensitive signaling pathways rather than acting independently. Epigenetic modifications can regulate the expression of antioxidant genes and signaling mediators involved in NF-κB, MAPKs, PI3K/Akt, AMPK, and Keap1–Nrf2 pathways, while oxidative stress can, in turn, alter epigenetic enzyme activity [90,91,92]. According to recent multi-omics studies, osteoclasts can coordinate stage-specific differentiation and bone-resorptive activity by integrating inflammatory, metabolic, and oxidative cues through bidirectional epigenetic–redox crosstalk [61,93,94]. These findings emphasize that epigenetic regulation is a dynamic interface linking osteoclast transcriptional programs to environmental stimuli.

### 7.4. Therapeutic Potential and Future Perspectives

Because epigenetic modifications are reversible, they represent promising therapeutic targets for treating oxidative stress-associated bone diseases. Preclinical studies have demonstrated that miRNA-based treatments, lncRNA-targeting strategies, histone deacetylase inhibitors, DNA methyltransferase inhibitors, and RNA therapeutics inhibit osteoclastogenesis by restoring redox homeostasis and reducing inflammatory signaling [95,96,97,98,99]. New strategies, such as targeted RNA delivery and CRISPR-based epigenome editing, have expanded the therapeutic potential of epigenetic interventions [100]. Nevertheless, most evidence remains preclinical, and further research is required to establish the clinical relevance and therapeutic efficacy of these epigenetic strategies.

## 8. Antioxidant Therapeutics Targeting Osteoclast Oxidative Stress

### 8.1. Natural Antioxidants and Phytochemicals

**Figure 2:**
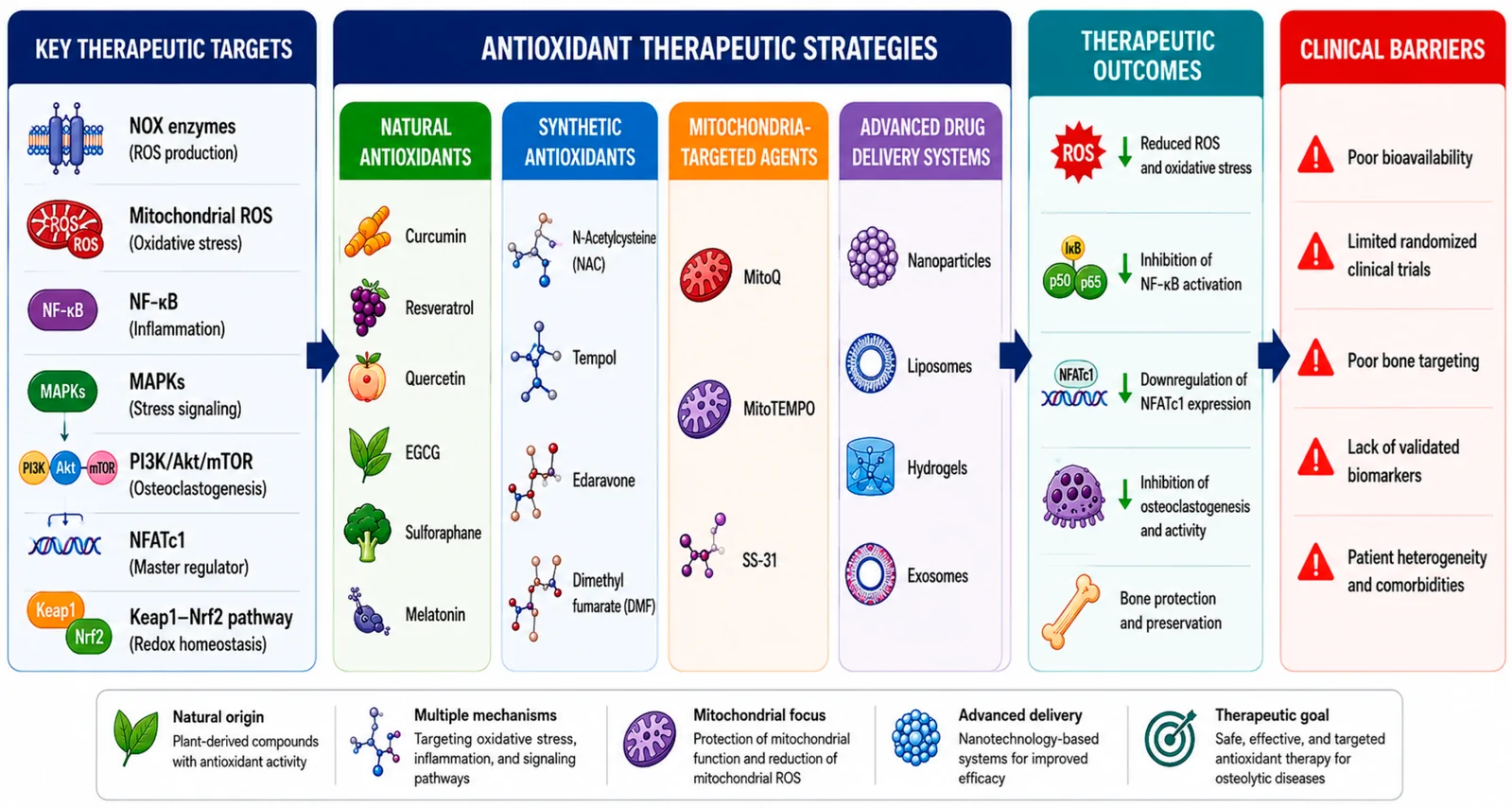
**Summary of antioxidant-based therapies aimed at reducing oxidative stress and osteoclast-mediated bone resorption**. Main therapeutic targets include ROS produced by NOX, mitochondrial ROS, NF-κB, MAPKs, PI3K/Akt/mTOR, NFATc1, and the Keap1–Nrf2 signaling pathway. Antioxidant therapies include natural antioxidants (curcumin, resveratrol, quercetin, EGCG, sulforaphane, and melatonin), synthetic antioxidants (NAC, tempol, edaravone, and dimethyl fumarate), and mitochondrial antioxidants (MitoQ, MitoTEMPO, and SS-31), as well as innovative drug delivery systems such as nanoparticles, liposomes, hydrogels, and exosomes. They may help to reduce ROS levels, attenuate redox-sensitive inflammatory and osteoclastogenic signaling, inhibit osteoclastogenesis and osteoclastic activity, and protect bone tissues. Nevertheless, clinical utility is limited by low bioavailability, insufficient bone-specific targeting, lack of validated biomarkers, and patient heterogeneity. Redox modulation may represent a more appropriate therapeutic strategy than ROS elimination.

Natural products constitute the largest class of antioxidant compounds investigated for osteolytic diseases owing to their pleiotropic biological activities (Figure 2, Table 3). In recent in vitro and animal studies, polyphenols, flavonoids, and other phytochemicals, such as resveratrol, curcumin, quercetin, EGCG, sulforaphane, luteolin, apigenin, and baicalein, have been shown to suppress osteoclast differentiation by reducing intracellular ROS, inhibiting NF-κB, MAPK, and NFATc1 signaling, and activating endogenous antioxidant pathways such as Keap1–Nrf2 [65,66,67,68,70,71,72,73,92,93,94,101,102,103]. Numerous phytochemicals exert multitarget protective effects against pathological bone resorption by enhancing mitochondrial function, reducing inflammatory cytokine production, and modulating metabolic pathways. Despite promising preclinical results, poor aqueous solubility, low oral bioavailability, rapid metabolism, and limited bone accumulation remain barriers to their clinical use.

### 8.2. Dietary and Endogenous Antioxidants

Skeletal redox homeostasis is supported by endogenous antioxidants and dietary micronutrients. Melatonin protects bones by directly scavenging ROS, improving mitochondrial function, and upregulating antioxidant enzymes such as GPx, catalase, and SOD [104]. Likewise, vitamin C supports collagen synthesis and osteoblast differentiation while reducing oxidative stress [105], and vitamin E protects membrane lipids from lipid peroxidation and suppresses inflammatory signaling [106]. Although experimental evidence consistently demonstrates the beneficial effects of antioxidants, clinical outcomes remain variable owing to differences in nutritional status, disease severity, dosage, and treatment duration.

### 8.3. Synthetic Antioxidants and Mitochondria-Targeted Therapies

Synthetic antioxidants with enhanced pharmacological characteristics have been developed to overcome the limitations of natural compounds. In recent preclinical investigations, N-acetylcysteine (NAC), edaravone, Tempol, dimethyl fumarate, MitoQ, MitoTEMPO, and SS-31 have been shown to reduce oxidative stress through complementary mechanisms, such as direct ROS scavenging, glutathione replenishment, activation of Nrf2 signaling, and selective targeting of mitochondrial ROS [107,108,109,110,111]. These agents may offer advantages in terms of pharmacokinetic properties, target specificity, and dosing control compared with some naturally occurring antioxidants. However, further research is required to determine long-term safety, optimal dosing regimens, and clinical efficacy.

### 8.4. Nanotechnology and Targeted Drug Delivery

Antioxidant compounds often have limited therapeutic efficacy owing to their poor bioavailability, rapid systemic clearance, and insufficient accumulation in bone tissue. Nanotechnology-based delivery platforms, including liposomes, polymeric nanoparticles, hydrogels, exosomes, and bone-targeting nanocarriers, may improve antioxidant delivery to bone tissue by enhancing drug stability, extending circulation time, improving bone-specific delivery, and reducing systemic toxicity [112,113]. Furthermore, ROS-responsive nanoparticles that release therapeutic agents in oxidative microenvironments have been actively studied for applications in bone regeneration, periodontitis, osteoporosis, and peri-implantitis [114,115]. These nanoparticles may provide opportunities for precise and localized antioxidant therapies.

### 8.5. Clinical Translation and Future Perspectives

Despite the encouraging preclinical evidence, relatively few randomized clinical trials have evaluated the efficacy of antioxidant therapies in osteoporosis, RA, periodontitis, and other osteolytic disorders. Clinical translation is hampered by heterogeneity in disease mechanisms, patient populations, dosage schedules, and treatment durations, as well as by the absence of standardized oxidative stress biomarkers. Future therapeutic strategies should prioritize biomarker-guided patient selection, targeted drug delivery, combination therapies, and precise modulation of redox signaling rather than indiscriminate ROS suppression. Additionally, the development of safe and efficient antioxidant therapies for oxidative stress-associated bone diseases is anticipated to be accelerated through the integration of single-cell and spatial multi-omics, artificial intelligence-assisted drug discovery, nanomedicine, and personalized medicine.

## 9. Current Limitations, Clinical Challenges, and Future Directions

Despite substantial progress in understanding the role of oxidative stress in osteoclast metabolism, several limitations have impeded the translation of experimental results into successful clinical treatments. Although numerous in vitro and animal studies have identified ROS as key regulators of osteoclast differentiation and bone resorption, many aspects of redox biology remain poorly understood. Furthermore, the development of targeted antioxidant therapies is hampered by the complexity of oxidative stress signaling, disease heterogeneity, and limited clinical evidence.

### 9.1. Mechanistic and Experimental Limitations

Despite considerable advancements in the understanding of redox-sensitive signaling pathways, the molecular thresholds that distinguish physiological ROS signaling from pathological oxidative stress remain poorly defined. Furthermore, the relative contributions of mitochondrial ROS, NADPH oxidase-derived ROS, ER stress, and iron-dependent oxidative pathways vary depending on the disease context and stage of osteoclast development [43,44,45,46,47,48,49,51,52]. Recent single-cell studies have revealed substantial heterogeneity among osteoclast precursors, suggesting that distinct osteoclast subsets may exhibit unique metabolic and redox programs that remain incompletely characterized [116,117]. Moreover, most mechanistic evidence is derived from rodent and cell culture models that often use varying experimental protocols and only partially recapitulate human skeletal diseases, thereby limiting the comparability and clinical relevance of findings across studies.

### 9.2. Challenges in Clinical Translation

Although preclinical studies have yielded encouraging results, the clinical application of antioxidant therapies remains limited. In experimental models, osteoclastogenesis has been effectively suppressed by natural antioxidants such as resveratrol, curcumin, quercetin, sulforaphane, EGCG, and melatonin, as well as synthetic antioxidants such as NAC, MitoQ, MitoTEMPO, and dimethyl fumarate [70,71,72,73,107,109,110]. However, the lack of well-designed randomized clinical trials, poor bioavailability, unfavorable pharmacokinetics, and limited bone-specific delivery remain major barriers to their clinical application.

Conventional anti-osteoporotic drugs could potentially affect the skeletal redox balance and serve as another clinical connection between oxidative stress and osteoporosis management. Low levels of estrogen have been shown to be associated with the weakening of the antioxidative defense system and with oxidative stress, whereas there is some evidence that estrogen maintains the activity of the thiol-dependent antioxidant system in bone under estrogen deficiency conditions [118]. Hormone replacement therapy in postmenopausal women is known to lower systemic oxidative stress markers, including plasma F2-isoprostanes and protein carbonyls [119,120]. Raloxifene therapy is able to alter the levels of oxidative stress-related biomarkers, such as malondialdehyde in the serum and erythrocyte superoxide dismutase activity; the significance of the latter finding, however, still needs to be clarified [121]. Statins might also affect the redox balance since the clinical studies using pravastatin and atorvastatin in hypercholesterolemic patients show conflicting results regarding the effects on plasma oxidative stress markers [122].

Furthermore, indiscriminate antioxidant therapy may compromise skeletal homeostasis because physiological ROS are necessary for normal bone remodeling [22,48,109]. This highlights the need to selectively modulate redox signaling rather than eliminate ROS indiscriminately.

### 9.3. Biomarker Discovery and Patient Heterogeneity

Another major challenge is the lack of reliable biomarkers that accurately reflect oxidative stress and predict therapeutic responses. Imaging and traditional bone turnover markers are the mainstays of current clinical evaluations; however, they offer little insight into intracellular redox status. Multi-omics studies have identified numerous transcriptomic, proteomic, metabolomic, and epigenetic candidates associated with osteoclast activity, but few have undergone extensive clinical validation [93,94,95,96]. Furthermore, oxidative stress levels and treatment outcomes may vary according to age, sex, genetic background, metabolic disorders, smoking, nutritional status, systemic inflammation, and microbiome composition, underscoring the necessity of biomarker-guided patient stratification.

### 9.4. Future Directions

Future research should integrate single-cell and spatial multi-omics, CRISPR-based functional genomics, advanced redox imaging, and artificial intelligence to identify stage-specific mechanisms that regulate osteoclast metabolism and oxidative stress [93,100,123]. Biomimetic platforms such as bone organoids and bone-on-a-chip models, along with targeted antioxidant delivery systems and biomarker-guided precision medicine, may enhance therapeutic specificity and accelerate clinical translation. Collectively, these multidisciplinary approaches may facilitate the development of personalized antioxidant therapies capable of selectively suppressing pathological bone resorption while preserving physiological skeletal remodeling.

## 10. Literature Search and Methodology

To identify relevant studies published up to June 2026, we conducted a comprehensive literature search using PubMed, Scopus, Web of Science, and Google Scholar. The search strategy combined Medical Subject Headings (MeSH) terms and free-text keywords, including osteoclasts, osteoclastogenesis, oxidative stress, reactive oxygen species, redox signaling, mitochondria, NADPH oxidase, Nrf2, NF-κB, MAPK, PI3K/Akt, AMPK, calcium signaling, epigenetics, mitophagy, bone remodeling, osteoporosis, periodontitis, rheumatoid arthritis, and antioxidant therapy. Peer-reviewed original research articles, meta-analyses, and systematic reviews were prioritized based on their relevance and methodological quality. Reference lists of relevant publications were manually screened to identify additional pertinent publications. Studies unrelated to osteoclast biology, oxidative stress, or musculoskeletal diseases were excluded. The selected literature was critically synthesized to provide an updated overview of the molecular mechanisms, metabolic regulation, antioxidant defense systems, therapeutic advances, current limitations, and future directions in the redox regulation of osteoclast biology.

## 11. Conclusions

Oxidative stress plays a key role in regulating osteoclast metabolism, differentiation, and bone-resorptive activity through coordinated interactions among redox-sensitive signaling pathways, mitochondrial function, metabolic reprogramming, and endogenous antioxidant defenses. Normal bone remodeling depends on physiological ROS, while excessive ROS accumulation disrupts redox homeostasis and contributes to pathological bone loss in osteoporosis, RA, periodontitis, and other osteolytic diseases. Although several promising antioxidant-based therapeutic approaches have been identified, clinical translation remains limited owing to inadequate clinical evidence, poor bioavailability, and an incomplete mechanistic understanding. Considering the central role of oxidative stress in pathological osteoclastogenesis, restoring redox homeostasis rather than indiscriminately scavenging ROS may represent a more appropriate therapeutic strategy for maintaining skeletal health. The continued integration of mechanistic research, emerging technologies, and well-designed clinical studies may facilitate the development of targeted antioxidant therapies for oxidative stress-associated bone diseases.

## Figures and Tables

**Table 1:** Major metabolic pathways regulating osteoclast differentiation and function.

Metabolic Pathway	Key Regulators/Enzymes	Primary Function	Role in Osteoclastogenesis	Therapeutic Implications
Glycolysis	GLUT1, HK2, PFKFB3, LDHA	Rapid ATP production and biosynthesis	Supports precursor proliferation, differentiation, multinucleation, and bone resorption	Glycolytic inhibitors suppress osteoclast formation
Mitochondrial oxidative phosphorylation (OXPHOS)	ETC complexes, PGC-1β, TFAM	Sustained ATP generation	Provides energy for mature osteoclast function and generates mitochondrial ROS	Modulation of mitochondrial function may reduce excessive osteoclast activity
Fatty acid oxidation	CPT1, PPARs	Alternative energy source	Supports mitochondrial metabolism during metabolic stress	Potential metabolic target in inflammatory bone diseases
Glutamine metabolism	GLS, GLUD, TCA cycle enzymes	Anaplerosis and redox balance	Maintains the tricarboxylic acid cycle (TCA) activity, biosynthesis, and antioxidant capacity	Glutaminase inhibition reduces osteoclast differentiation
Metabolic plasticity	AMPK, mTOR, HIF-1α	Adaptation to nutrient and oxygen availability	Coordinates metabolic remodeling during physiological and pathological conditions	Emerging target for precision metabolic therapy

**Table 2:** Key sources of ROS and redox-sensitive signaling pathways in osteoclasts.

ROS Source	Major Mediators	Activated Signaling Pathways	Biological Effects	Associated Diseases
NADPH oxidases	NOX1, NOX2, NOX4	NF-κB, MAPKs, and NFATc1	Initiates RANKL-mediated ROS production and osteoclast differentiation	Osteoporosis, arthritis, and periodontitis
Mitochondria	Electron transport chain	PI3K/Akt, AMPK, and mTOR	ATP production, mitochondrial ROS generation, and metabolic remodeling	Aging and osteoporosis
Endoplasmic reticulum stress	PERK, IRE1α, ATF6	UPR and NF-κB	Calcium dysregulation and oxidative stress	Inflammatory bone diseases
Iron metabolism	Fenton reaction, ferroptosis	Lipid peroxidation pathways	ROS amplification and oxidative damage	Iron overload-associated bone loss
Inflammatory cytokines	TNF-α, IL-1β, IL-6, IL-17	NF-κB and MAPKs	Enhance ROS generation and osteoclast activation	Rheumatoid arthritis and periodontitis
Hypoxia	HIF-1α	HIF signaling, mitochondrial adaptation	Alters metabolism and increases ROS production	Periodontitis, bone defects
Environmental/metabolic factors	Aging, diabetes, smoking	Multiple redox pathways	Chronic oxidative stress and enhanced bone resorption	Osteoporosis and diabetic bone disease

**Table 3:** Antioxidant therapeutics targeting oxidative stress-induced pathological bone loss.

Antioxidant/Strategy	Primary Target	Major Mechanism	Evidence	Clinical Status
Curcumin	NF-κB, Nrf2	Suppresses ROS and inflammatory signaling	In vitro and animal studies	Limited clinical evidence
Resveratrol	SIRT1, Nrf2	Antioxidant, anti-inflammatory, and mitochondrial protection	In vitro and animal studies	Early clinical studies
Quercetin	NF-κB, MAPKs	ROS scavenging and inhibition of osteoclastogenesis	In vitro and animal studies	Limited
EGCG	NF-κB, RANKL signaling	Reduces oxidative stress and inflammation	In vitro and animal studies	Limited
Sulforaphane	Keap1–Nrf2	Activates endogenous antioxidant defenses	Animal studies	Emerging
Melatonin	Mitochondria, antioxidant enzymes	Improves mitochondrial function and redox homeostasis	Animal and clinical studies	Promising
N-acetylcysteine (NAC)	Glutathione	Restores intracellular antioxidant capacity	Experimental and clinical studies	Widely used antioxidant
MitoQ/MitoTEMPO	Mitochondria	Selective scavenging of mitochondrial ROS	Animal studies	Experimental
Dimethyl fumarate	Nrf2	Activates endogenous antioxidant signaling	Preclinical studies	Approved for other indications
Nanoparticle-based delivery	Bone tissue	Improves bioavailability and targeted delivery	Preclinical studies	Emerging technology

## Data Availability

No new data were created or analyzed in this study. Data sharing is not applicable to this study.

## Abbreviations

The following abbreviations are used in this manuscript:

M-CSF	Macrophage Colony-Stimulating Factor
RA	Rheumatoid Arthritis
RANK	Receptor Activator of Nuclear Factor-κB
RANKL	Receptor Activator of Nuclear Factor-κB Ligand
NFATc1	Nuclear Factor of Activated T Cells 1
ROS	Reactive Oxygen Species
NADPH	Nicotinamide Adenine Dinucleotide Phosphate
Keap1	Kelch-Like ECH-associated Protein 1
Nrf2	Nuclear Factor erythroid 2-related Factor 2
TRAP	Tartrate-Resistant Acid Phosphatase
CTSK	Cathepsin K
DC-STAMP	Dendritic Cell-Specific Transmembrane Protein
CTR	Calcitonin Receptor
ATP	Adenosine Triphosphate
HK2	Glycolytic Enzymes Like Hexokinase 2
PFK	Phosphofructokinase
PKM2	Pyruvate Kinase M2
GTT1	Glucose Transporter Type 1
OXPHOS	Oxidative Phosphorylation
FAO	Fatty Acid Oxidation
TCA	Tricarboxylic Acid
AMP	Adenosine Monophosphate
AMPK	AMP-Activated Protein Kinase
mTOR	Mechanistic Target of Rapamycin
HIF-1α	Hypoxia-Inducible Factor-1α
NOXs	NADPH Oxidases
ER	Endoplasmic Reticulum
NF-κB	Nuclear Factor Kappa B
UPR	Unfolded Protein Response
CREB	cAMP Response Element-Binding Protein
TRAF6	Tumor Necrosis Factor Receptor-Associated Factor 6
TAK1	Transforming Growth Factor-β-Activated Kinase 1
EGCG	Epigallocatechin Gallate
AREs	Antioxidant Response Elements
HO-1	Heme Oxygenase-1
NQO1	NADPH Quinone Oxidoreductase-1
SODs	Superoxide Dismutases
GPxs	Glutathione Peroxidases
Trx	Thioredoxins
Prxs	Peroxiredoxins
ncRNAs	Non-Coding RNAs
miRNAs	MicroRNAs
ceRNAs	Competing Endogenous RNAs
lncRNAs	Long Non-Coding RNAs
circRNAs	Circular RNAs
NAC	N-acetylcysteine
